# Using theoretical engagement to understand workplace learning across contexts—Bringing worlds apart together

**DOI:** 10.1111/medu.15481

**Published:** 2024-08-06

**Authors:** Francisco M. Olmos‐Vega, Renée E. Stalmeijer

**Affiliations:** ^1^ Pontificia Universidad Javeriana Bogotá Colombia; ^2^ School of Health Professions Education Maastricht University Maastricht Netherlands

## Abstract

The pivotal importance of workplace learning (WPL) within health professions education has elevated its understanding and improvement to a major research priority. From a sociocultural learning theory perspective, WPL is inherently situated and context‐specific. This means that the health care settings in which (future) health care professionals are trained will impact how and what is learned. However, to what extent is the research performed thus far transferable across professional contexts, cultures and borders? To what extent has WPL research sufficiently addressed the contextual characteristics of WPL to enable the evaluation of its transferability? To what extent have methodological and theoretical approaches enabled the building of understanding across contexts?

We propose that heightening the transferability of WPL research as well as opening up the conversation to more diverse WPL contexts, settings and cultures will require mapping context and theoretical engagement. To explore what theoretical engagement may afford to our understanding of the influence of context on WPL, we use two theories: Landscapes of Practice and Figured Worlds. These theories with sociocultural groundings provide concrete lenses to understand the interplay between the individual and the context. We conclude with implications for research and practice and advocate for more attention to research practices that may deepen our understanding and heighten the transferability of workplace learning research.

## Abstract 2

Dive into the pivotal role of context in #WorkplaceLearning within health professions education while discovering the power of theoretical engagement.

We are worlds apart, but we share the same dreams.

S.V. Mastison

## 1 WORKPLACE LEARNING AND THE IMPORTANCE OF CONTEXT

Workplace learning (WPL) holds great significance in medical education as it facilitates the development of clinical competencies and the formation of professional identities through active engagement in real‐world practices.^1^, ^2^, ^3^ Theoretically, WPL is considered situated, as it occurs in specific situations or contexts where the knowledge is intended to be applied.^4^ Lave and Wenger originally defined situated learning as a dynamic learning process occurring through legitimate peripheral participation in communities of practice (CoPs). This process, which is fundamentally social and embedded in everyday activities, involves progressively increasing participation in a specific context^5^ and has seen considerable use in the field of health professions education (HPE).^6^ In the case of HPE, trainees learn through participating in real‐life health care contexts under the supervision and guidance of more experienced health care professionals. Due to the diverse nature of these health care contexts, outcomes of WPL can vary and transitioning to new contexts may result in partially having to relearn specific competencies.^7^ For example, executing surgical procedures will differ based on available medical equipment, which antibiotics are prescribed is determined by local antibiotic resistance and how primary care is organised will determine care for patients with chronic conditions.^8^, ^9^ Contextually bound practices and their variations can also include how doctors and patients communicate, the predetermined hierarchy between attending physicians, trainees and allied health care professionals and the extent to which interprofessional collaboration is encouraged.^9^, ^10^ WPL is then the result of the deliberate interaction between trainees and the possibilities and boundaries of the context in which they are embedded.^11^ Some of these things are relatively easy to learn (which equipment is available, antibiotics resistance and care organisation), while others take time, such as understanding each other's role within the health care team, how the work flows, how and when to participate in it and how the physical location influences these processes.^12^ This means that competence is in itself contextual and that certain things will need to be (re)learned and taught again and again.^13^, ^14^, ^15^ Considering that undergraduate and postgraduate students train in a wider variety of clinical settings than ever before,^16^, ^17^ ranging from high‐complexity urban hospitals to community‐based medical centres in rural areas, and adding geographical^18^ and cultural variations,^19^ a vast range of contexts must be considered to understand and improve WPL. Research on WPL has therefore started to explore how trainees' interactions and engagement with the health care contexts directly impact their learning and how these settings impact their access to learning opportunities and, consequentially, their learning outcomes.^10^, ^20^, ^21^

There is no dispute that context matters in WPL; yet, rather than provide detailed accounts about contextual characteristics, researchers seem to shy away from sharing that which makes their contexts unique. This ‘fear of context’ seems to originate from the post‐positivist value that research should be generalisable across settings. Although some scholars agree on the futility of trying to generalise findings from educational research,^22^ the aim of HPE research is nevertheless to move the field forward and improve how we prepare future health care professionals for practice. But how can we acknowledge the influence of context in our research while also promoting its transferability to other, potentially very different contexts? We argue that moving WPL research forward and heightening its transferability can be guided by clearly defining and discussing context^11^, ^23^ as well as through theoretical engagement.^24^

## 2 DEFINING AND CAPTURING CONTEXT

Few studies in medical education,^23^ and in WPL specifically,^25^ explicitly describe or define their context, often focusing only on isolated factors such as physical settings or clinical disciplines. In response to this issue, Bates and Ellaway proposed a clear definition of context that can be used in HPE research,^23^ namely, that context is a constantly evolving system shaped by the dynamic and intersecting patterns of locations, practices, patient profiles, learning interactions, culture, society and the unforeseeable interactions among these elements.^23^ Schrewe et al. built on the work of Bates and Ellaway and offered a model called the contextual curriculum to explore how context influences WPL in clinical settings.^11^ The contextual curriculum underscores that learning in medical education is intrinsically linked to the specific settings in which it takes place. By acknowledging and comprehending the various contextual patterns—patients, practice, educational, physical, health care system and culture—researchers, educators and learners are enabled to better understand how these elements shape learning outcomes and identify and capitalise on different contexts' unique learning opportunities.^11^ Ultimately, engaging deeply with the contextual curriculum cultivates more adaptable, context‐sensitive health care professionals who are better equipped to address the diverse needs of patients and communities. Reporting these elements in WPL research and incorporating them in practice enhances the relevance and applicability of educational strategies and ensures that medical training remains responsive to the dynamic and varied landscapes of health care practice.

Contrary to worries of generalisability, deliberately reporting contextual details is an important practice in heightening the extent to which findings from one study can be readily applied in another but similar context.^26^ This practice is commonly understood as transferability and is an important quality criterion for qualitative research.^26^, ^27^ However, Stalmeijer et al. recently argued that more transferability practices and dimensions should be considered.^24^ Beyond providing sufficient detail for readers to evaluate the applicability of research findings in their own settings,^28^ transferability could also be heightened by presenting research in such a way that its findings resonate with the reader.^29^ A third dimension that is specifically relevant in research across contexts is heightening the transferability of research through theoretical engagement.^30^ Transferability as theoretical engagement involves using cumulative research findings to advance our understanding of a theoretically informed phenomenon.^31^ This can be done by connecting the findings to an existing theory or by proposing a new model or theory.^30^ As such, transferability at the theoretical level addresses how the findings of a study can be used to build on our existing knowledge and understanding of the world across contexts.

To exemplify how we can better understand WPL across contexts, we will be applying context mapping as well as theoretical engagement to a case. Using previously published empirical work,^10^ we will use two theoretical frameworks, Landscapes of Practice (LoP)^32^ and Figured Worlds (FW),^33^ as well as the lens of context mapping to demonstrate the impact that cross‐contextual differences may have on our understanding of WPL. Before we do this, in keeping with the principles of reflexivity, we share how our personal and interpersonal characteristics may have impacted how we have approached the issue of context in WPL.

## 3 ADDRESSING AUTHORS' PERSONAL AND INTERPERSONAL REFLEXIVITY

How context impacts research into WPL, as well as seeking ways to heighten the transferability of this area of research, speaks to us personally as cross‐cultural collaborators in the field of WPL. We met when R. E. S. was assigned to be F. O. V.'s Master thesis supervisor while he was working on a Master's in HPE at Maastricht University. F. O. V. went on to do a PhD at Maastricht University under the guidance of R. E. S. and two other Dutch supervisors. We continued our collaborations due to our shared research interests in WPL, sociocultural learning theories, qualitative research methodology and optimising HPE and health care practice through education and research. In this section, we provide a personal reflexivity to give the readers an insight into our personal positioning with the HPE landscape, highlighting the nature of our relationship and how our personal histories might have shaped how we approach the arguments in this paper (see Box 1).

Box 1 **Authors' personal reflexivity.**

**Table:** 

Francisco Olmos‐Vega, MD, MHPE, PhD (F. O. V.) I am an anaesthetist and assistant professor from Bogotá, Colombia. My interest in WPL is deeply rooted in my role as an active supervisor of medical students during my anaesthetist duties. This interest has been a continuous thread throughout my academic journey, beginning with my doctoral studies and extending into my current roles as a teacher and master thesis supervisor. Navigating the intricate landscape of WPL within the realm of health professions education has been a pivotal aspect of my scholarly pursuits. However, this journey has not been without its challenges, particularly in trying to publish my work. Attempting to disseminate my research in the global north requires a delicate balance—a balance between accentuating cultural elements that can seamlessly transcend borders and mitigating those that may not easily align with the expectations of a Western audience. While adapting my research to meet certain expectations can undoubtedly facilitate its dissemination within academic circles, it also necessitates careful consideration of how such adaptation may inadvertently diminish the unique cultural perspectives that I believe hold relevance on a global scale. Finding this equilibrium, I believe, is essential for preserving the integrity of my research while fostering a meaningful dialogue in the broader academic community. Applying theory to my research has served me well in this endeavour, while also being the crucial reason to write this paper.	Renée E. Stalmeijer, MSc, PhD (R. E. S.) I was born and raised in the South of the Netherlands. I studied Educational Sciences at Maastricht University (MU). After attaining my Master's degree, I stayed on at MU as a junior researcher combining a role in programme evaluation of the medical curriculum with a PhD project on evaluating clinical teaching through cognitive apprenticeship. Early on, learning as a social‐constructivist process as well as post‐positivist research values were drilled into me. During my PhD, I started to explore other, more sociocultural theoretical frameworks and was exposed to qualitative research methodology.The School of Health Professions Education at MU enabled me to start supervising Master and PhD students from across the globe, ranging from Colombia to Japan and Indonesia to Canada.During these interactions, I was struck by struggles of transferability—for example, clinical supervision models developed in the Global North could not be transferred to the Japanese context without addressing Confucianist values.^18^ Furthermore, I was struck by the difficulty of convincing Global North editors of the value of research done in the Global South and that a dance ensued about what level of detail should be provided in the methods section and word count, something I struggled less with when publishing with colleagues from the Global North.

While writing and discussing this current manuscript, we were amazed to experience how, over the years, what we valued in research on WPL had started to align. Our narratives as cross‐cultural collaborators reveal the complexities and nuances of navigating WPL within HPE. Our combined experiences have led us to an approach to HPE research that underscores the importance of theoretical frameworks in articulating and preserving contextual elements in issues related to HPE. This approach not only enriches the quality but also enhances the global relevance of our work. In our work, we experienced how the thoughtful application of theory bridges cultural divides, ensuring that research remains robust and resonant across diverse contexts.

## 4 USING TWO THEORETICAL FRAMEWORKS TO ENSURE THEORETICAL TRANSFERABILITY

In this section, we will explore two different yet complementary sociocultural theoretical frameworks—LoP^32^ and FW^33^—to identify relevant contextual features that might impact how learners navigate and learn from clinical practice settings and to enhance the transferability of WPL research across contexts. We chose these theories as a sociocultural standpoint that enables a more nuanced comprehension of contexts as fluid and flexible systems while exploring the interplay between such contexts and the learners that engage with them.^34^, ^35^ These are certainly not the only theories suitable for exploring context in WPL research. However, as Philibert et al. argued,^36^ although the sociocultural lens on learning has gained wider acceptance in HPE research, its selective and narrow application has led to certain limitations, including the field's appreciation of the low impact that informal learning has on trainees' competence and professional development. For example, in a scoping review, O'Brien and Battista found that the situated learning theory has been used inconsistently and often superficially in HPE literature.^6^ Therefore, attending to context to improve the transferability of research findings entails engaging with theory on a deeper level: either utilising theory consistently to guide research design and data analysis or developing, modifying or extending a theory to establish a new one.^37^ This section will present and explore two theoretical frameworks using one of the published articles we co‐authored as a case. In Box 2, the reader will find the details of the study case, including key findings, implications for practice, contextual elements and conclusions. To enable further understanding of these two theoretical frameworks, we also present a comparative table with key aspects of each theory (see Table 1).

Box 2 **Summary of the article *Disentangling residents' engagement with communities of clinical practice in the workplace*. Olmos‐Vega, F. M., Dolmans, D. H., Guzmán‐Quintero, C., Echeverri‐Rodriguez, C., Teunissen, P. W., & Stalmeijer, R. E.^10^**

This study examined how medical residents engaged with communities of clinical practice (CoCP) when starting new clinical rotations. Using a constructivist grounded theory approach and semi‐structured interviews supported by the Pictor technique, the authors interviewed 13 residents from different disciplines and levels of training at a major high‐complexity urban medical centre in Bogotá, Colombia.

Key findings:

- *Complex processes of engagement*: Residents underwent recurring and intertwined processes when entering new CoCPs, including exploring the alignment of their goals with those of the CoCP, identifying key CoCP members and understanding how those members could assist their integration into community practices.
- *Varied participation trajectories*: Residents' intentions influenced how they engaged with CoCPs. Those seeking a central role within the CoCP (apprentices) aligned their goals closely with the community's expectations, whereas those aiming for a peripheral role (sojourners) negotiated their participation based on their own objectives.
- *Negotiation of roles*: Alignment between a resident's goals and CoCP expectations influenced their role clarity and dynamics. Misalignment often resulted in vague roles and tensions, leading to limited learning opportunities.
- *Importance of interprofessional actors*: Nurses, respiratory therapists and other health care team members significantly impacted residents' learning experiences. A collaborative team environment fostered better alignment and learning opportunities.

Implications for practice:

- *Maximising learning opportunities*: Aligning workplace affordances with various resident trajectories could reduce missed learning opportunities.
- *Flexibility in learning goals*: Supervisors could assist residents in identifying their intended roles and adjusting their goals during rotations.

Contextual elements:

- *Patients*: The study took place in a tertiary urban medical centre, which takes care of patients with various levels of complex conditions, but mostly high‐complexity ones.
- *Practice*: The study included residents from varied disciplines and clinical settings, which entailed different practices within the same hospital: from settings with patients with acute and complex conditions that need the coordination of multiple interprofessional actors (as the ICU) to settings with patients with more chronic conditions with fewer interprofessional actors working in the same location (such as a respirology unit).
- *Educational*: Residencies in Colombia follow sequential clinical rotations, which are scheduled in blocks of a minimum of 2 months and a maximum of 6 months. Each residency programme has a fixed number of rotations within its domain but also includes rotations outside its discipline. During clinical rotations, residents carry out various clinical tasks under supervision, following a model that gradually increases their responsibility and autonomy.
- *Physical*: The residents that participated in the study took part in various rotations that included different physical settings within the same medical centre, such as the ICU, hospitalisation ward, emergency room, labour room and outpatient clinic.
- *Health care system*: Residents in Colombia play a pivotal role in patient care, acting as direct caregivers in clinical settings. This central responsibility underscores their essential contribution to the overall functioning and quality of medical centres within the health care system, highlighting their significant engagement in direct patient care and clinical decision making.
- *Culture*: Although there are initiatives related to high‐quality accreditation standards that promote interprofessional collaboration, the practice culture has a highly hierarchical, traditional structure with an emphasis on intraprofessional learning.

Conclusion: The study concluded that understanding how residents negotiated their roles within CoCPs could help optimise learning in clinical settings. The engagement of the entire health care team was crucial for maximising residents' learning potential in diverse clinical environments.

**TABLE 1:** Comparison between Landscapes of Practice and Figured Worlds theories.

	Landscapes of Practice	Figured Worlds
Main focus	Interconnectedness of communities within a landscape	Construction of identities within cultural and historical contexts
	Social and cultural dynamics shaping learning trajectories	Reciprocal relationship between individual and their social contexts
Affordances	Identifies modes of participation: apprentice, sojourner, tourist and broker	Explores how positionality, power, agency and improvisation influence learning
	Highlights knowledge and resource sharing across communities	Reveals how individuals interpret and make sense of cultural contexts
	Examines how learning trajectories within a landscape of practice lead to unique knowledgeability	Examines negotiation of roles and identities among actors
Limitations	Less focussed on individual interpretations and meaning‐making	Less sensitive to broader, interconnected social structures
	May overlook positionality and power influence to learning experiences	May overlook how learning trajectories might impact students learning

### 4.1 Landscapes of Practice

LoP theory emphasises the social and cultural aspects of learning and knowledge creation in specific contexts.^32^ It recognises the interconnectedness between individuals, their activities and their physical and social environments. In this sense, learning is not solely an individual process but is shaped by the social and cultural practices of a particular community or group. This theory highlights the dynamic nature of learning and knowledge creation as individuals and communities continually negotiate and transform their practices and understandings.^32^ LoP is an evolution of the previously described theory of CoPs^5^ and was created as a response to the evolving complexities of learning in diverse and interdisciplinary contexts. LoP was specifically developed to acknowledge varying communities that may be active within a particular field of practice (like health care and medical trainee development), the boundaries between them and the broader social and cultural context in which they are situated.^32^ The fluid and permeable boundaries between CoPs within an LoP allow for knowledge and resource sharing across communities.^38^ When applied to health care, LoP highlights the interprofessional nature of health care and the complexities related to varying CoPs, like those of physicians, nurses, physiotherapists and nutritionists, aiming to provide optimal care.^39^ LoP foregrounds the importance of interprofessional collaboration and would suggest that better preparing health care trainees for future practice requires their engagement with the entire health care team.^40^ See Box 3 for a description of the key LoP concepts.

Box 3 **Key LoP concepts.**

Elements of LoP theory that can enhance the theoretical transferability of WPL include *modes of identification*, the *roles* of trainees within the landscape and the concept of *knowledgeability*.

1. *Modes of identification*: Trainees build their identities in the LoP through three modes^32^, ^38^, ^40^:
  - *Imagination*: Trainees envision themselves within the landscape, helping them understand their place and role.
  - *Alignment*: Trainees and communities align their practices, goals and values, creating a shared understanding.
  - *Engagement*: Trainees actively participate in specific communities of practice (CoPs) within the landscape.
2. *Roles within LoP*: Trainees engage with CoPs in four distinct roles.^15^, ^41^, ^42^
  - *Apprentices*: They learn and develop skills within a specific CoP, usually with guidance from mentors or experienced practitioners.
  - *Sojourners*: These trainees temporarily enter a CoP to gain knowledge, often as outsiders or newcomers.
  - *Tourists*: They visit a CoP for a short time, typically as observers or passive participants.
  - *Brokers*: They connect different CoPs within the landscape, facilitating the exchange of knowledge and resources between communities. Brokers could include trainees as well as established CoP members.
3. *Knowledgeability*: This concept refers to the expertise and understanding that trainees develop through their journey in the LoP. It is a concept specifically developed through the LoP theory. It encompasses both individual and collective knowledge and the ability to apply it in practice.^7^, ^32^ Knowledgeability evolves through continuous learning, reflection and interaction within the LoP. It also involves building relationships that result in trainees being recognised as reliable sources of information or legitimate providers of services. In health care, this means understanding and collaborating effectively with others and aligning professional practices within the broader landscape.^40^, ^43^ For example, a third‐year medical resident in an intensive care unit (ICU) might develop knowledgeability by actively engaging with the multidisciplinary team, including nurses, respiratory therapists and senior physicians. Through daily rounds, they discuss patient cases, contribute to decision making and learn from the expertise of different team members. Over time, the resident becomes proficient in managing complex patient cases, understands the workflow of the ICU and is recognised by peers and supervisors as a knowledgeable and reliable practitioner. This resident's knowledgeability is demonstrated by their ability to integrate medical knowledge, practical skills and effective communication within the team, ultimately improving patient care outcomes.

LoP theory helps us understand situated learning from the trainee's perspective as they navigate a complex landscape. Additionally, contrasting and comparing trainees' journeys through culturally diverse landscapes helps us better understand the situated nature of WPL. For example, in our study, residents assumed the role of either an *apprentice* or a *sojourner* when entering a new rotation, depending on how they configured their modes of identification within that rotation. Both types of residents featured a high *engagement* mode but differed in their *alignment* and *imagination* while engaging with the LoP; *apprentices* imagined themselves as an integral part of that LoP and, therefore, aligned their preferences and goals with those of the LoP they entered. Conversely, sojourners did not imagine themselves as an integral part of the LoP and, consequently, had to negotiate their alignment with their different members. Residents then developed a unique *knowledgeability* that reflected the various roles they assumed while navigating the LoP. For instance, a general surgery resident who functions as an apprentice during an intensive care unit rotation will develop a knowledgeability that is characterised by a clinically oriented approach to patient care. This resident, guided closely by mentors and immersed in hands‐on clinical practice, gains a deep understanding of intensive care unit procedures and patient management. In contrast, a peer who acts as a sojourner in the same rotation will develop a different form of knowledgeability, perhaps gaining a broader but less intensive understanding of critically ill patients due to their temporary and less integrated role in that specific CoP. As LoPs are culturally shaped, different contexts may produce diverse landscapes, resident types and knowledgeabilities. The constant push to engage residents in clinical tasks in Colombia's health care system and the privileging of clinical rotations within specific disciplines—both contextual features of the landscape in which we conducted the study—may explain why our study did not find tourist or broker resident types. Explorations across postgraduate learning contexts, settings and cultures that applied LoP theory could, for example, help us identify circumstances where residents could assume the tourist or broker role and understand how such situations could impact their knowledgeability. Additionally, these explorations could help us understand how organisations promote varying resident roles. In this sense, the theory can not only help us identify relevant contextual features that could impact the way learners choose to engage with a situated landscape but it could also facilitate the transfer of knowledge across contexts through theoretical engagement.

### 4.2 FW theory

FW theory is a sociocultural theory that explores how social context and historical experiences shape the construction of individuals' identities^33^ (see Box 4 for an explanation of key FW concepts). The theory also emphasises the mutuality and reciprocity between the individual and their social context, which means that individuals actively shape their identity and the social context in which they exist.^44^ As a result, the concept of FW has significant implications in interpreting social and cultural constructs. Within this realm, people assign meaning to specific events and actions performed by distinctive actors, ultimately shaping their understanding of the world.^33^ Understanding a specific FW is crucial in comprehending how individuals interpret and make sense of their cultural context. Therefore, it holds incomparable value when it comes to understanding the influence of context upon actors while also providing affordances to explore differences and similarities across contexts.^19^, ^45^ Given that identity construction, participation in real practice and learning are inseparable processes in sociocultural theories, FW theory can aid in expanding our understanding of WPL.^5^, ^20^ As we stated before, the situated nature of WPL influences trainees' competency development, and FW theory gives us specific affordances to understand the interplay between the individual trainee and the workplace context. For example, in surgery and internal medicine FWs, the practices inherent to each specialty not only dictate the content and methods of teaching but also profoundly shape residents' learning experiences.^19^ In surgical training, learners experience more direct and personal coaching in developing their psychomotor skills, risking their reputation in a hands‐on, closely watched setting. On the other hand, in internal medicine, trainees work on understanding patient cases more theoretically, trying to align their understandings with those of their teachers.^19^ The roles of clinical teachers and learners are not just functionally distinct in different FWs; they are deeply rooted in the cultural milieu of their respective fields. Consequently, the unique contexts in which trainees learn significantly influence the dynamics, timing and frequency of social interactions, the topics explored and the pedagogical approaches employed. For instance, context greatly influences how residents learn health care management (HCM) skills during their clinical rotations; FWs that promote interprofessional collaboration, have supervisors who model how to incorporate HCM tasks into daily activities and give residents the necessary tools to complete them favour HCM skills learning.^46^

Box 4 **Key FW concepts.**

An FW is a sociocultural concept that refers to a socially and culturally constructed realm where individuals participate in various activities, interact with others and develop specific identities. The term, introduced by cultural anthropologist Dorothy Holland and her colleagues, emphasises the dynamic interplay between personal agency and social structures within these worlds.^47^ Elements of FW theory that help us understand the context of WPL include *agency*, *improvisation*, *positionality*, *power* and *discourse*.^48^

1. *Agency*: Agency is the ability of individuals to make choices and take actions to achieve their goals using the tools available in the FW. It is not fixed; it emerges through interactions within social and cultural environments. However, it is constrained by the resources provided by the FW.^33^
2. *Improvisation*: Improvisation involves combining existing tools or resources within a social context to create novel and appropriate responses to tensions or contradictions. It showcases agency by navigating conflicting expectations within the FW.^49^
3. *Positionality*: Positionality refers to a person's social location within the FW, determined by factors like gender, race or educational rank. It shapes experiences and perspectives by providing or restricting access to cultural tools, helping individuals figure out their roles and construct their identities.^50^
4. *Power*: Power is the ability to influence others' actions, either through formal authority or informal influence. Positionality grants varying levels of power within the FW, affecting individuals' capacity to act and interact.^33^
5. *Discourse*: Discourse describes how individuals express their thoughts, beliefs, attitudes and identities within a particular FW.^11^, ^33^ Influenced by social contexts, discourses can be multiple and contradictory. Individuals may align with or contest these discourses, using them to construct identities and respond to specific circumstances.^51^, ^52^

FW theory, by contrast with LoP theory, can help us understand situated learning from the perspective of the multiple actors that participate in a given context. The concept of FW sheds light on how individuals interpret and make sense of the world around them; it helps to explain how people come to understand and assign meaning to events and actions. For example, in our study case, health care team members constantly negotiate residents' participation on each rotation. Using the FW framework, we can explore the results from the perspectives of these health care allies. We can ponder, for example, how a specific allied health care professional uses their *agency* to interact and collaborate with the new resident and in which circumstances they decide to *improvise* to either facilitate or hinder their participation in patient care. Additionally, a health care ally's *positionality* could give them access to privileged knowledge regarding how things work in a given scenario. This knowledge is not readily available to residents, so it gives the ally specific *power* over them; the actor can either wield their power to withhold the information from the residents, hindering their participation in patient care, or they can decide to share their knowledge and integrate the resident more easily into daily practice. By exploring these choices in‐depth, we can understand how to enable residents' learning through interprofessional interactions. However, different contexts can give differential positionality and power to the various actors in that specific FW, influencing their agency and improvisation possibilities. Explorations across contexts that applied FW theory could, therefore, help us identify circumstances where health care allies hold less privileged positionalities and the impact of such variation in how they interact and influence residents' training. Additionally, we could also explore how this power is distributed to other actors and how this influences the residents' learning within a specific health care team. Again, by engaging with this theory, we can provide more contextual information within research projects while expanding the evidence base for WPL research across geographically diverse settings.

## 5 CONTRASTING LoP AND FW AS LENSES FOR WPL RESEARCH

In summary, LoP and FW theories provide comprehensive and nuanced frameworks for understanding the influence of culture and context on WPL, particularly within HPE. LoP offers a perspective that emphasises the interconnectedness of communities within a practice landscape and how social and cultural dynamics shape learning trajectories. It identifies modes of participation such as apprentice, sojourner, tourist and broker and highlights knowledge and resource sharing across communities. LoP also examines how learning trajectories within a landscape lead to unique knowledgeability, though it may overlook individual interpretations and the influence of positionality and power.

Conversely, FW focusses on exploring the reciprocal relationship between individuals and their social contexts. It delves into how positionality, power, agency and improvisation influence learning, revealing how individuals interpret and make sense of cultural contexts. FW examines the negotiation of roles and identities among actors, but it is less sensitive to broader, interconnected social structures and may overlook how learning trajectories impact students' learning. By combining both perspectives, these theories enable a dynamic and culturally responsive understanding of WPL. This approach is crucial for adapting educational strategies to diverse and evolving health care environments. Using theories like LoP and FW to study WPL enriches our understanding and builds a cumulative knowledge base that transcends cultural and contextual boundaries within HPE (Table 1).

## 6 IMPLICATIONS FOR FUTURE RESEARCH

Recognising that education and learning are deeply contextual is fundamental to enhancing the value of research in HPE.^20^, ^53^ Acknowledging the impact of the nuances of context in clinical practice on learning trajectories and interactions pushes us to consider the rich tapestry of context in our studies.^15^, ^53^ This awareness prompts a more thorough examination of transferability in research, challenging us to critically assess how findings from one context can inform and enrich another.^11^, ^23^ We can start by exploring and understanding how already published WPL models and processes can be effectively translated to diverse contexts.

Expanding our research lens to include a global perspective necessitates the inclusion of diverse geographical and professional contexts, particularly from areas often overlooked in HPE research.^54^ We enrich our understanding of WPL environments by using theoretical frameworks effectively; theory is the thread that could help us weave the tapestry of WPL understanding.^55^, ^56^ Comparative studies informed by theory across various health professions could further illuminate the unique and shared aspects of professional development within different clinical and disciplinary settings. Although we selected LoP and FW specifically, this goal could also be achieved by a wide range of other theories, including theories such as Activity Theory or Socio‐Materialism. Whatever theory is selected, what is essential is using the theory with sufficient depth.^37^ This might entail utilising it as a lens to guide research design, data collection, interpretation and synthesis. For example, key concepts can be used to inform the construction of questioning guides or inductive codes.^48^ Theoretical engagement then provides a common frame of reference, making it easier for readers worldwide to understand and relate to the research findings.

To move forward, the HPE field must acknowledge the need for more detailed information to assess the transferability of research and move beyond its abstract notions of context.^23^, ^57^ This shift will require detailed, thoughtful descriptions of learning settings and an understanding of the practitioners within these environments. By envisioning a global audience and striving for rich, in‐depth context descriptions, we not only heighten the relevance of our research but also its applicability. These suggestions will shed light on the practical implications of educational and clinical frameworks, guiding the development of more supportive learning environments across the health professions.

## 7 IMPLICATIONS FOR PRACTICE

WPL within clinical settings is a multifaceted and dynamic process.^58^ HPE research on WPL has concentrated on optimising interactions among clinical teachers, students and health care professionals to enhance learning outcomes. Through this focus, HPE researchers developed targeted recommendations to improve the effectiveness of WPL.^59^, ^60^ However, to truly refine learning in the workplace, all participants—educators, learners, health care allies and patients—must truly comprehend the full extent of their context. Such understanding goes beyond the superficial acknowledgement of the environment; it involves acknowledging how each unique setting influences the learning process. This deeper awareness can catalyse the critiquing and revising of established practices; it could also aid clinical teachers in determining the transferability of the practical implications that WPL research has generated to diverse contexts. Continuous professional development programmes can provide clinical teachers with the tools necessary to achieve this task. By fostering this comprehensive understanding of context, we might pave the way for a smoother learning process that actively challenges entrenched norms and promotes a workplace culture that prioritises student learning and patient safety. This cultural shift is not merely beneficial but critical for the evolution of WPL and the overarching aim of health care: delivering safe, high‐quality patient care.

## 8 REFLEXIVE EPILOGUE

Through our (F. O. V. and R. E. S.) shared discussions, we have once again been confronted with the need for and complexity of research across contexts. We have found value in embracing situated and theoretically informed research to better grasp HPE's research challenges. This is not to say that more cognitivist and post‐positivist lenses do not have a place in this field; on the contrary, only by combining the best of various worldviews will we enable the deep understanding needed to improve and optimise WPL within the clinical context. In short, there is more work to be done. First, by stimulating more cross‐cultural collaborations, and second, by a stronger focus on heightening the transferability of the work within our field, be it situated within the Global South or Global North. Even though we may be worlds apart, we share the same dreams. Let us bring them to life.

## AUTHOR CONTRIBUTIONS

**Francisco M. Olmos‐Vega:** Conceptualization; writing—review and editing; writing—original draft. **Renée E. Stalmeijer:** Conceptualization; writing—review and editing; writing—original draft.

## CONFLICT OF INTEREST STATEMENT

The authors do not have any conflict of interest to declare.

## ETHICS STATEMENT

This type of article did not need approval from our local ethics review board.

## Data Availability

Data sharing is not applicable to this article as no datasets were generated or analysed during the current study.
